# Tissue-specific Tregs in cancer metastasis: opportunities for precision immunotherapy

**DOI:** 10.1038/s41423-021-00742-4

**Published:** 2021-08-20

**Authors:** Laura A. Huppert, Michael D. Green, Luke Kim, Christine Chow, Yan Leyfman, Adil I. Daud, James C. Lee

**Affiliations:** 1 Division of Hematology/Oncology, Department of Medicine, University of California, San Francisco, San Francisco, CA USA; 2 Department of Radiation Oncology, University of Michigan School of Medicine, Ann Arbor, MI USA; 3 Veterans Affairs Ann Arbor Healthcare System, Ann Arbor, MI USA; 4 University of California, San Francisco School of Medicine, San Francisco, CA USA; 5 Penn State College of Medicine, Hershey, PA USA; 6 Parker Institute for Cancer Immunotherapy, San Francisco, CA USA

**Keywords:** Tissue Tregs, Immunotherapy, Organ-specific tolerance, Metastasis, Cancer, Immunosuppression, Immunosurveillance

## Abstract

Decades of advancements in immuno-oncology have enabled the development of current immunotherapies, which provide long-term treatment responses in certain metastatic cancer patients. However, cures remain infrequent, and most patients ultimately succumb to treatment-refractory metastatic disease. Recent insights suggest that tumors at certain organ sites exhibit distinctive response patterns to immunotherapy and can even reduce antitumor immunity within anatomically distant tumors, suggesting the activation of tissue-specific immune tolerogenic mechanisms in some cases of therapy resistance. Specialized immune cells known as regulatory T cells (Tregs) are present within all tissues in the body and coordinate the suppression of excessive immune activation to curb autoimmunity and maintain immune homeostasis. Despite the high volume of research on Tregs, the findings have failed to reconcile tissue-specific Treg functions in organs, such as tolerance, tissue repair, and regeneration, with their suppression of local and systemic tumor immunity in the context of immunotherapy resistance. To improve the understanding of how the tissue-specific functions of Tregs impact cancer immunotherapy, we review the specialized role of Tregs in clinically common and challenging organ sites of cancer metastasis, highlight research that describes Treg impacts on tissue-specific and systemic immune regulation in the context of immunotherapy, and summarize ongoing work reporting clinically feasible strategies that combine the specific targeting of Tregs with systemic cancer immunotherapy. Improved knowledge of Tregs in the framework of their tissue-specific biology and clinical sites of organ metastasis will enable more precise targeting of immunotherapy and have profound implications for treating patients with metastatic cancer.

## Introduction

In immuno-oncology, recent progress and efforts spanning a century have led us to the present state of cancer immunotherapy [1–3]. Today, cancer drugs that harness the immune system can completely and eradicate solid tumors long-term to achieve cures of even in stage IV cancer. In cancer, nondeleterious “passenger” mutations accumulate in tumor cells over time, and these “altered-self” neoantigens increase immunogenicity, which can lead to antitumor immune recognition [4,5]. Checkpoint inhibitors (CPIs) take advantage of this preexisting immunity and induce cytotoxic T cell attacks on tumor cells. Microsatellite instability-high (MSI-H) cancers and melanoma are among the cancers with the highest tumor mutation burden (TMB), which correlates with their profound and durable response to CPIs [6,7]. However, with the excepts of melanoma and MSI-H cancers, fewer than fifteen percent of cancer patients receive this level of benefit from immunotherapy [6–8]. Unexpectedly, in certain cancers, such as non-small cell lung cancer (NSCLC), CPIs do not reach a level of high efficacy or long-term response despite a high TMB, and the median overall survival (OS) is usually less than 20 months. The variability in CPI response across cancer types can be attributed to various intrinsic and extrinsic mechanisms of resistance in the tumor. However, in melanoma, MSI-H, kidney, and certain other malignancies where CPIs have shown potent efficacy, clinical evidence suggests that the presence of metastases in specific organs, such as the liver, is associated with reduced immunotherapy response and survival [9–12]. These observations suggest the possibility that tissue site-specific factors may impact antitumor effector function, regardless of the level of immune recognition or T cell reinvigoration by CPIs [13,14].

Interestingly, there is tissue site variability in CPI efficacy and autoimmune toxicities [15–17]. Within each tissue, there are potential differences in thresholds and determinants for immune activation because of differences in biological function and involvement in host defense. Tissues resistant to therapy exhibit an inability to break through this tolerance despite a checkpoint blockade. In cases of toxicity, tissue-specific patterns in loss of tolerance likely tilt the immune balance toward excessive activation and aberrant autoimmune attack. The clinical differences and putative mechanisms that govern tissue-specific autoimmune CPI toxicity constitute an area of active investigation and have been recently reviewed by Young et al. and Kang et al., respectively [17,18]. There is a growing awareness that tumors within organs are populated with tissue-adapted immune cell subsets, evolutionarily driven by their particular biological function within the organ. Recent work by our group and others revealed organ-specific tolerance mechanisms that not only shape local antitumor immunity but also exert a significant influence on a distant site, potentially through a coordinated tolerogenic effect [19–21]. Currently, neither tumor-intrinsic nor tumor-extrinsic resistance pathways are clearly understood in the context of the specialized, tissue-specific microenvironment [22]. It is likely that future therapeutic strategies to overcome resistance mechanisms will need to reconcile tissue-specific factors to be successful.

**Fig. 1:**
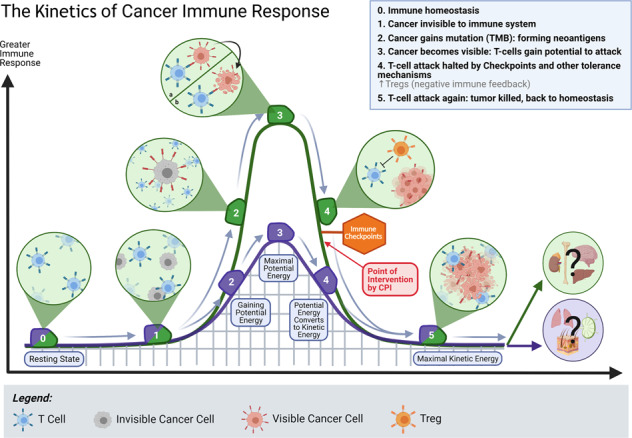
The kinetics of cancer immune responses. Tissues may have different thresholds of immune activation and tolerance. (0) At a steady state, without infection, injury, or tumor, tissues are in immune homeostasis and are populated by tissue-specific resident immune cells. (1) Cancer cells are often initially invisible to local immune surveillance. Due to the lack of neoantigens and inflammatory signals, these cells can be perceived as “self” by tissue-resident immune cells. (2) Tumor cells gain mutations over time, forming neoantigens, making them more visible to local immune surveillance. However, different tissues have different thresholds of immune activation, a system that has been adapted to support their function. (3) When the activation threshold is met, tumor cells are visible to immune surveillance and subject to T cell attack. In this context, different tissues may exhibit different tolerance levels for maximal immune activation, resulting in variable limits of “potential energy” that is generated. (4) In some tissues, as T cells attack the tumor, the “kinetic energy” may never reach adequate levels because the assault is halted by potent tissue-adapted negative immune feedback mechanisms such as immune checkpoints and Tregs. CPIs can reverse immunosuppression, but only in a minority of cases. Additional intervention, such as tissue-specific targeting of Tregs, is needed to induce full antitumor immunity. (5) After induction of local antitumor immunity, when the “kinetic energy” of the response is depleted, the tissue returns to a homeostatic state. In some tissues, the cancer is eradicated at this point, but in other tissues, such as the liver, cancer cells typically remain, causing Tregs to repeatedly reinforce homeostasis, ultimately causing a wound that does not heal.

Specialized immune cells known as regulatory T cells (Tregs) reside in the systemic circulatory system and within all tissues. They are known as the master controllers of self-tolerance and function to maintain immune homeostasis and coordinate the suppression of excessive immune activation to prevent autoimmune responses. The observation that these potent suppressive T cells are also ubiquitous in tumors, or “altered-self” tissues, was first described by Robert North in 1980 [23]. However, although Tregs constitute an immune cell subset that has been among the most extensively studied in oncology for decades, the successful targeting of Tregs for cancer immunotherapy has been elusive because these cells are seldom studied in the context of their critical tissue-specific properties. Emerging evidence suggests that Tregs demonstrate remarkable adaptability to their local environment and facilitate immune homeostasis through highly specialized tissue-specific pathways [24]. After the effective elimination of pathogenic threats, the evolutionarily evolved immune system immediately restores quiescence and prevents further harm [25]. After tumors acquire neoantigens to induce an immune response, potent immune suppressors, such as Tregs, are typically upregulated. Tolerogenic Treg mechanisms have coevolved with specialized biological functions in each organ, and different strategies are required to achieve tumor immunity (Fig. 1). Therefore, a major challenge for effective precision cancer immunotherapy of metastatic disease is the decoding and understanding of the tissue-adapted, multilayered, regulatory processes that have explicitly evolved to protect the various tissue sites coopted in cases of metastatic cancer.

With this review, we aim to improve the understanding of Tregs in the framework of their tissue-specific function and secondary impact on metastasis immunity in hopes of advancing more precise targeting of immunotherapy for metastatic cancer. We begin with a primer on the latest understanding of how Tregs modulate tissue repair and wound healing and how these pathways, shared across tissues, contribute to cancer metastasis. Then, we focus on the tissue-specialized role of Tregs in several clinically relevant cancer metastasis sites, including the liver, bone, skin, lungs, brain, adrenal gland, and lymph nodes (LN). For each organ, we highlight findings from recent work describing the specialized biology of Tregs and their implications for tumor immunity and immunotherapy within that tissue. Finally, we close with a summary of recent advances in several translational treatment strategies that combine the targeting of tissue-specific Tregs with systemic cancer immunotherapy.

## Tregs in tissue repair and cancer metastasis

There is mounting evidence suggesting that, in addition to peripheral tolerance, Tregs are critical regulators of tissue repair and wound healing across tissues. This critical function is highly evolutionarily conserved, as even zebrafish FoxP3-expressing Tregs are recruited to injured tissue [26]. In mammalian skin, inflammation following injury is necessary to protect against infection, but excessive inflammation and subsequent myeloid cell infiltration may impair wound healing [27]. After skin injury, activated CD25, CTLA-4, and ICOS^hi^ Tregs are recruited from secondary lymphoid organs (SLOs) [26,28]. Mice depleted of Tregs in the five days following wounding displayed significantly greater wound closure time than wild-type mice, with the Treg-depleted mice exhibiting increased granulation tissue and size of the resulting eschar, suggesting the importance of early Treg action following skin wounding [29]. Skin-resident Tregs express the transcription factors IRF4 and GATA-3, which suppress T_H_2 immune responses. A number of inflammatory mediators, such as IL-18, IL-33, and thymic stromal lymphopoietin, are released after tissue damage. These molecules stimulate Treg-induced expression of amphiregulin (AREG), a member of the epithelial growth factor (EGF) family that promotes epithelial regeneration and keratinocyte differentiation [30,31]. AREG-dependent production of transforming growth factor-β (TGF-β) was found in an acute tissue injury-induced pericyte-to-myofibroblast differentiation and proliferation postinjury model [31–33]. Studies with mice depleted of Tregs prior to injury further suggest that this process is modulated by the growth and differentiation factor activin [29]. Tregs directly suppress the inflammation during tissue repair by altering the wound cytokine profile. Upon Treg depletion in a mouse model of skin injury, the number of conventional IFNγ- or IL-17-producing αβ T cells increased within the wound and were associated with the significant elevation of cytokine IL-4, which impairs cutaneous wound healing by repressing the expression of fibronectin, an extracellular matrix protein that promotes keratinocyte migration and wound closure [29,34].

In muscle tissue repair, Tregs modulate macrophage differentiation. Following muscle injury, the population of proinflammatory Ly6c^hi^ and CD11b^+^Gr1^-^ myeloid mononuclear cells was substantially increased, but by the fourth-day postinjury, the population of these cells was decreased, and the population of anti-inflammatory Ly6c^lo^ cells was increased [35] Treg ablation in post-myocardial infarction (MI) mouse model of acute skeletal muscle injury resulted in the failure of myeloid cell lines within the wound infiltrate to switch from expressing a proinflammatory M1 phenotype to an anti-inflammatory M2 phenotype, impairing cardiac muscle remodeling and post-MI recovery [35,36]. Finally, injured skeletal muscle in Treg-depleted mice lacked regenerative muscle fibers and exhibited decreased muscle progenitor cells. AREG expressed by Tregs mediates muscle regeneration by enhancing myosatellite cell differentiation [35].

Tumor cells cause local destruction and inflammation as they proliferate within the tissue, and some are even capable of tumor cell-intrinsic inflammatory signaling [37]. The repeated injury and inflammation induced by unchecked tumor growth drive a vicious cycle that becomes difficult to stop. From the perspective of the tumor microenvironment (TME) and the metastatic niche, the multilayered Treg mechanisms for tissue repair can be coopted to promote cancer progression because they simultaneously prepare the local “soil” for tumor regeneration and growth while protecting the “seed” against immune rejection. Thus, there has been great interest in understanding the role of Tregs in cancer growth and proliferation within the TME [38]. Tregs have been extensively studied in the peripheral blood and immune infiltrates of different cancers, and a higher Treg-to-effector T cell ratio within tumor tissue is associated with worse patient prognoses in many tumor types, including melanoma, pancreatic ductal adenocarcinoma [39], NSCLC [40], ovarian cancer [41,42], glioblastoma [43], non-Hodgkin lymphoma [44], and others.

**Fig. 2:**
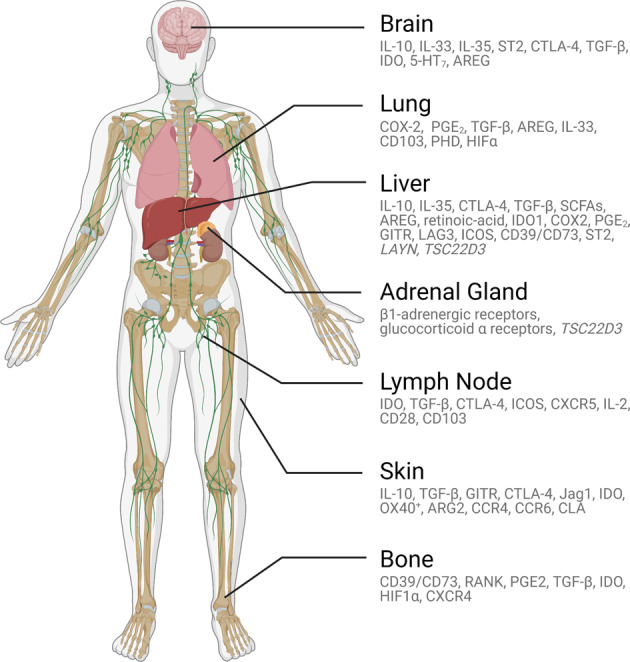
Tissue-specific regulatory T cell (Treg) mediators. Tregs in different organs serves distinctive functions via different mediators. In addition to maintaining self-tolerance, Tregs have adapted organ-specific specialized functions that support tissue but contribute to ineffective antitumor immunity. From top to bottom: Tregs in the brain express neurotransmitter receptors, respond to microglial cells to dampen autoimmunity, and facilitate neuronal injury repair, including in ischemic injury repair. Tregs in the lungs can express oxygen-sensing proteins, mediate tolerance to inhaled aeroallergens and type 2 immunity against commensals, and support tissue repair and remodeling after airway infections. Tregs in the liver can respond to microbiota-derived metabolites, mediate dietary oral tolerance, regulate immunity to gut commensals, support tissue repair and regeneration, and possibly maintain hematopoietic stem cell quiescence during fetal development. Tregs in the adrenal gland express glucocorticoid receptors and may respond to stress response signaling. Tregs in lymph nodes maintains tolerance by controlling T follicular helper cells and B cells. Tregs in the skin dampen autoimmunity, regulate tolerance to commensals, mediate wound healing, and support hair growth. Finally, Tregs in the bone maintain hematopoietic stem cell quiescence and facilitate osteogenesis.

Research on the link between the direct role of Tregs in cancer metastasis is ongoing and active. Tumor-infiltrating Tregs generally express higher levels of cell surface molecules associated with T cell activation, such as CD25, CTLA-4, PD-1, LAG3, TIGIT, ICOS, 4-1BB, OX-40, and GTFR [45]. These cells are thought to suppress effector T cells directly, contribute to the metabolic disruption of effector T cells, and modulate the maturation and function of dendritic cells required for effector T cell activation [46]. Tregs are also thought to play a role in promoting tumor metastases by facilitating tumor dissemination, immune evasion, and preparing metastatic foci [47]. For example, increased Treg frequencies are associated with a greater risk of metastasis in many cancers, including breast, ovarian, prostate, lung, thyroid, gastric, colorectal, and skin cancers [47–49]. Higher levels of tumor-infiltrating Tregs are associated with increased tumor size, while increased proportions of peripheral blood Tregs are associated with clinical stage, pathological differentiation, and LN metastasis [50]. We summarize key mediators of Treg biology (Fig. 2) and highlight the latest research on these processes in the context of their specialized, tissue-specific functions within each clinically relevant site of cancer metastasis.

## Tregs in lung metastasis

Experimental studies have shown that Tregs have a central function in maintaining immune homeostasis in healthy lungs. This finding is not surprising, as the respiratory tract has evolved to interface with the environment and has an impressive alveolar surface area of ~70 m^2^ in adult humans that can be subjected to constant exposure to environmental antigens during respiration, making control of local immune homeostasis a tightly regulated process [51]. An acute inflammatory response after infection or injury that remains unresolved can lead to an immediately life-threatening impairment of alveolar oxygen exchange, making immunoregulation critical [52]. Under physiological conditions, Tregs are thought to mediate tolerance of inhaled innocuous environmental aeroallergens, type 2 immunity in response to commensals, and subsequent IgE production [26,51]. The alarmin IL-33, produced by lung cells after exposure to allergens, induces mast cells to produce IL-2, which expands the Treg population within the lungs [53]. Failure of Treg suppression of the chronic immune response to aeroallergens has been attributed to airway tissue remodeling and is a hallmark of asthma [51]. In addition, Treg expression of the EGF family member AREG is critical in pulmonary tissue repair after airway infections and is apparently IL-33-dependent but TCR-independent [54].

Lung cancer is the leading cause of cancer-associated deaths worldwide, with 85% of cases due to NSCLC and 15% of cases due to SCLC. Exposure to tobacco smoke, environmental carcinogens, and inflammatory lung disease are all risk factors, and it is thought that these inflammatory states alter inflammatory cytokine levels, oxidative stress markers, and immune cell composition. Despite reports suggesting that the lungs are sites of improved CPI response in metastatic disease, immune escape plays a major role in the development and progression of primary lung cancer and metastasis, and it is thought that Tregs are recruited to the tumor tissue and facilitate tumor cell escape from immunological surveillance [15,55]. Specifically, Tregs can induce immunosuppression through contact-independent mechanisms such as the sequestration of IL-1 and the production of soluble immunosuppressive molecules such as TGF-β, IL-10, prostaglandin E2, IL-10, and galectin-1 and contact-dependent mechanisms such as the expression of cytotoxic T-lymphocyte-associated protein-4 (CTLA-4), programmed cell death-1 (PD-1), programmed death-ligand one (PD-L1), lymphocyte-activation protein-3 (LAG-3), and neuropilin 1 (NRP1) [56–58].

Preclinical work has suggested that Tregs play an important role in the early stages of lung tumor development. In murine models of mutant Kras-driven lung adenocarcinoma, tumorigenesis was dependent on Tregs, and Kras transgenic mice that were deficient in FoxP3^+^ Tregs developed 75% fewer lung tumors than wild-type mice [59]. Tregs are also thought to play a role in the TME during the development and progression of lung cancers. Murine models of lung adenocarcinoma have revealed that Tregs likely play a role in inhibiting CD8 T cell-mediated antitumor immunity, as depletion of Tregs has been associated with tumor cell death and elevated levels of IFNγ, granzyme A, granzyme B, and perforin in infiltrating CD8 T cells [60]. In SCLC cell lines, Treg generation is induced by CD4 T cells through the production of IL-15, further supporting the notion that tumor cells can manipulate Tregs to their advantage [61].

In addition to enabling tumor growth and development, Tregs have also been suggested to promote the development of metastatic tumor foci. In a study of 23 patients with NSCLC, Treg levels in the peripheral blood increased with tumor stage and were highest in patients with metastatic disease [62]. Interestingly, the Restifo group showed that the lung may be a tumor-permissive organ due to the tissue-specific expression of oxygen-sensing prolyl-hydroxylase (PHD) proteins in a preclinical model and that changes in extracellular oxygen tension induced Treg quantity and function through an increase in PHD proteins and a local reduction in HIF1α, which correlated with increased intrapulmonary metastasis [63].

Prognostically, there is a growing body of literature suggesting that high Treg infiltration predicts the risk of recurrence and clinical outcomes. For example, patients with SCLC with higher ratios of Tregs in the tumor infiltrate exhibited worse OS [61]. In NSCLC, elevated levels of cyclooxygenase-2 (COX-2), which transforms Tregs, have been associated with significantly worse recurrence-free survival than observed in patients with low COX-2 tumor expression [64]. Similarly, in a study of 64 patients with stage I NSCLC, patients who had a higher proportion of tumor Tregs relative to tumor-infiltrating lymphocytes showed a significantly higher risk of recurrence [65].

There has also been interesting in evaluating whether the frequency of Tregs can predict response and resistance to immunotherapy in lung cancer. In a recent study by Koh et al., the frequency of circulating Tregs was assessed one week after anti-PD-1 immunotherapy was administered to patients with NSCLC, and the data were correlated with clinical outcomes such as progression-free survival (PFS) and OS. This group found that the frequency of FoxP3^+^ Tregs and TGF-β predicts the response to anti-PD1 immunotherapy: The cohort with a high frequency of circulating Tregs 1 week after receiving anti-PD1 therapy had a significantly longer PFS (1.7 months vs. 7.9 months, *P* = 0.008) and OS (4.6 months vs. 12.3 months, *P* = 0.01) than those with a low frequency of circulating Tregs. In contrast to Tregs, which are thought to enable tumor proliferation, Th17 cells are CD4^+^ T helper cells that prevent the expansion of malignant cells in the TME and modulate antitumor immune responses [66]. In lung cancer, tumors with a higher ratio of Tregs to Th17 cells were shown to be associated with more aggressive biological responses and rapid tumor proliferation [67]. The Treg/Th17 ratio has also been studied in pleural fluid and blood [68]. Collectively, these data suggest that specialized Tregs are important for promoting the development, progression, and metastasis of lung cancer and may be clinically relevant for evaluating lung cancer prognosis and an important target for treating lung metastasis.

## Tregs in bone metastasis

The skeletal system, including the bone marrow (BM), is a unique microenvironment. It is the primary site of hematopoiesis and is critical for the production of innate and adaptive immune cells [69]. Therefore, skeletal tissue where immune responses need to be tightly regulated and controlled to prevent autoimmune reactions. For all cancers, bone is consistently reported to be among the top three most common organs for metastases [70]. Metastasis to the bone is correlated with a poor response to immunotherapy across several cancer types [12]. Data from the Checkmate 057 study, a phase III trial comparing nivolumab to docetaxel as second-line therapy in NSCLC, patients with bone involvement were less responsive to treatment [71]. For prostate and breast cancers, bone is the most common metastatic site, occurring in 70–80% of cases in patients with advanced disease [69,72]. Immunotherapy has shown minimal efficacy against both diseases, with response rates to PD-1-based monotherapy generally less than 10% [73,74]. Recently, the Sharma group reported data from a phase II trial (NCT02985957) that demonstrated that in patients with prostate cancer metastasized to bone, the CPI response rate was only 4% [75]. Since the prostate and breast are the two most common cancers, there is an urgent need to understand barriers to immunotherapy response.

Given the above, there is reason to speculate that the bone microenvironment may uniquely suppress immunotherapy. Tregs are essential for the maintenance of homeostasis in this dynamic environment. In the BM niche, potent specialized CD150^hi^ Tregs facilitate the maintenance of hematopoietic stem cell (HSC) quiescence and longevity through adenosine generated by exonucleotidases CD39 and CD73, and BM Tregs are present in significantly higher levels than LN Tregs under physiological conditions [76]. Their presence can be disrupted by blocking CXCR4, which is required for Treg re-entry into the BM, and Treg-specific ablation of CD39 was shown to abrogate the Treg suppressive effect. Notably, even allo-HSCs are protected by BM Tregs, and allo-HSCs were rapidly lost after the depletion of Tregs in a mouse transplant model [77]. Furthermore, Tregs are known to tilt the balance between osteoclasts and osteoblasts toward osteogenesis by suppressing osteoclast differentiation and function, a process that may favor the formation of the osteoblastic bone lesions seen in prostate cancer [78].

In metastatic disease, Tregs further migrate to the tumor-associated BM via the CXCR4/CXCL12 signaling pathway, and RANK ^+^ DCs induce Treg cell expansion [78]. In a preclinical model of castration-resistant prostate cancer (CRPC), CPIs significantly increased intratumoral T_h_1 cells and enhanced survival of a subcutaneous model but failed to elicit a response or generate T_h_1 cells in a bone model. In this study, TGF-β mediated an increase in Tregs in the TME, with an associated increase in T_h_17 cells. Combination therapy with anti-CTLA-4 plus anti-TGF-β significantly reduced Treg and increased T_h_1 cell levels [75]. In a mouse breast cancer model, overexpression of COX2 resulted in an increase in bone metastasis and increased Treg recruitment to the tumor without accompanying myeloid-derived suppressor cells (MDSCs). Elevated levels of PGE2 in tumor cells led to the recruitment of Tregs, and blockade using an anti-PGE2 antibody or genetic suppression of COX2 expression in tumor cells reduced bone metastases [79]. In another breast cancer bone metastasis model, inhibition of the CXCL12/CXCR4 axis in combination with IDO1 expression reduced Treg levels and bone metastasis [80].

The RANK–RANKL signaling pathway is of particular interest. In bone, this pathway is involved in controlling osteoclastogenesis and bone resorption. Interestingly, Tregs are for producing RANKL-expressing metastatic breast cancer cells, expressing fourfold more RANKL mRNA than Tconvs; blockade of this pathway can reduce the frequency of pulmonary metastasis [81]. It is thought that the bone microenvironment generates a prometastatic niche feedback loop since both osteoclasts and Tregs produce more RANKL in the presence of tumors, which induces further bone resorption. In turn, the resulting resorption of bone lacuna by osteoclasts causes local acidification of the microenvironment to activate TGF-β, which generates more Tregs [75]. The evidence further suggests that TCR triggering of BM tumor antigen-specific Tregs, but not Tconvs, induces tumor cell egression from the BM into the peripheral blood and tumor tissue, mediated by emigration receptor S1P1 and homing receptor CCR2, providing a plausible mechanism for bone metastasis reducing systemic antitumor immunity [82]. Finally, the BM is a relatively hypoxic environment at baseline, and hypoxia has been shown to increase Treg cell number and function in a HIF1α-dependent manner and promote bone metastasis [83]. A targeted combination approach will be needed to address bone metastasis and its potential influence on systemic antitumor immunity. Indeed, the Sharma group reported that their model experiments with a therapy consisting of anti-CTLA-4 plus anti-TGF-β showed a significantly increased frequency of antitumor T_h_1 cells with a concomitantly decreased frequency of Tregs in bone metastasis [75]. However, the anti-RANKL antibody seems to be a more effective inhibitor of bone metastasis and has been shown to be even more effective than bisphosphates [84].

## Tregs in liver metastasis

The liver immune system is highly specialized to adapt to several notable hepatic functions. First, a significant portion of blood from the gastrointestinal tract drains via the portal vein [85]. Dietary antigenic load taken up by Peyer’s patches and lamina propria enters the bloodstream via the portal vein, reaching the liver before entering the systemic circulation. As many consumed antigens are harmless, the liver is essential in preventing immune over-reactivity and contributes to antigen-specific oral tolerance [86]. As the largest internal organ in the body, the liver receives approximately 1.5L of blood from the circulatory system per minute via the portal vein as well as the hepatic artery [85]. Thus, in addition to innocuous dietary and commensal gut antigens, the liver comes into contact with blood-borne bacteria, viruses, parasites, and cancer cells. The mammalian liver is also unique in its ability to regenerate following partial liver resection, which requires a well-coordinated immune cell program to maintain optimal pro- and anti-inflammatory mediators [87]. Interestingly, in mammals, the liver serves as the main multilineage hematopoietic organ during fetal development [88]. Therefore, during development, homeostasis, and injury, the liver is a multifunctional immunoregulatory organ capable of orchestrating well-controlled immune responses.

The complex immune functions of the liver require highly specialized adaptive and innate immune cells and have long been of interest to immunologists. Immune cell subsets within the liver have been extensively reviewed [85,89]. A striking feature of liver histology is the abundance of liver-resident cell types that contribute to Treg generation, including but not limited to Kupffer cells (KCs), liver sinusoidal endothelial cells (LSECs), hepatic stellate cells (HSCs), hepatocytes, and dendritic cells (DCs) [89]. Tregs are essential for hepatic tissue immune homeostasis. The depletion of Tregs during development can result in increased hepatic type 1 inflammation, autoimmune hepatitis, and subsequent metabolic disorders [26]. Several well-studied mediators are frequently highlighted in the context of Treg maintenance of immune quiescence in the liver. IL-10 is a suppressive cytokine that modulates Treg activity, and IL-10 secreted by Tregs contributes to their suppressive function [90]. For example, hepatic DCs and KCs can induce and expand Tregs through IL-10, while Tregs themselves are the predominant source of IL-10 in the gut [26,91]. CTLA-4 is a critical regulatory protein constitutively expressed by Tregs and is thought to be key to the critical mechanisms of Treg suppression through its competition with CD28 for CD80/86 binding or induced reduction in APC CD80/86 expression [92]. TGF-β is abundant in the liver microenvironment and is both secreted and internalized by Tregs. LSECs, HSCs, and KCs produce TGF-β, which can facilitate the generation of liver Tregs via multiple mechanisms, including the conversion of Tconvs into Tregs [93]. Interestingly, activated liver Tregs are able to mediate systemic and extrahepatic antigen-specific tolerogenesis, a feature uniquely exploitable for autoimmune therapy purposes in non-liver diseases, such as type-1 diabetes and multiple sclerosis [94]. Finally, emerging data suggest that certain specialized Treg mediators are adapted to liver function in gut immune homeostasis. Microbiota-derived metabolites such as short-chain fatty acids can modulate and induce Tregs, potentially through the modification of histone deacetylase activity, suggesting epigenetic regulation [95]. Fat-soluble vitamin A is converted into retinoic acid (RA) by HSCs and LSECs, and RA induces gut-homing receptors on Tregs and enhances their suppressive function [96]. Interestingly, the brain/CNS was recently shown to modulate Tregs through a vagal neural arc that involves the liver; specifically, the “liver-brain-gut arc” modulated Treg numbers in a colitis model. The vagal sensory afferent nerves from the liver provide signals to the brainstem and mediate feedback to the parasympathetic nerves and enteric neurons, which ultimately results in RA synthesis by colonic APCs [97].

In the metastatic cancer setting, consistent with their capabilities in tissue homeostasis, liver-derived Tregs are reportedly able to suppress both local and extrahepatic antitumor immunity. For cancers where CPIs are effective and routinely used, such as melanoma, lung cancer, urothelial and kidney cancers, we and others have shown that liver metastasis is associated with a significantly lower rate of response to immunotherapy (<25%) and lower survival [9,12]. The mechanism of immunosuppression by Tregs in the context of liver metastasis is an area of active research, and much of the available knowledge has been derived from observations of primary liver cancers such as hepatocellular carcinoma (HCC), not the systemic immune impact of liver metastases. In HCC, the quantities of Tregs within the tumor and the blood are increased, and higher numbers of Tregs are often associated with worse outcomes for patients with either primary or metastatic liver tumors [98]. Histologically, there may also be a correlation between higher tumor grade and poorly differentiated liver tumors [99]. In prostate cancer liver metastasis, an association was found between increased Tregs and local IDO1 expression and the loss of the tumor suppressor gene PTEN in the patient tissue samples [49]. Phenotypically, data suggest increased activation status and potency of Tregs associated with liver tumors. For example, in metastatic colorectal cancer (mCRC) with liver metastasis, increased COX-2^+^ Tregs have been associated with increased blood prostaglandin E_2_ (PGE_2_), and they were found to correlate with decreased TNFα and IFNγ on CD3 T cells and unfavorable outcomes [100]. In HCC and mCRC with liver metastases, Tregs expressed elevated levels of GITR, LAG3, ICOS, CD39, and CTLA-4 [98]. Blockade of these inhibitory surface proteins led to improved antitumor immunity, often when blocked in combination [101]. The cytokines IL-10, IL-35, and TGF-β1 have been associated with enhanced Treg-mediated suppression of antitumor immunity in HCC [102–104]. Whole transcriptome analysis of Tregs revealed that the genes encoding layilin (*LAYN*) and glucocorticoid-induced leucine zipper (*TSC22D3)* are upregulated in Tregs associated with liver tumors [20,105].

Finally, two recent studies investigated the mechanism of liver metastasis suppression of extrahepatic tumor immunity using a syngeneic immunocompetent murine tumor model. In one report, liver metastases created a systemic “immune desert” with reduced antitumor CD8 T cells via FasL-mediated clonal deletion of tumor antigen-specific T cells by CD11b^+^ suppressive monocytes [21]. In contrast, CD11b^+^ suppressive monocytes were also found to be abundant in extrahepatic tumors in mice bearing liver tumors, but this abundance was dependent on an associated increase in CTLA-4- PD-1-, and ICOS-high Tregs. Depletion of highly activated Tregs with Treg-depleting anti-CTLA-4 antibody was able to reverse liver metastasis-mediated systemic immunosuppression [20].

## Tregs in skin metastasis

The skin, a part of the integumentary system, is considered the largest and most exposed organ in the human body [106]. With an estimated surface area of 1.8 m^2^ in adults and containing twice the number of T cells than the circulatory system, at approximately 20 billion cells, it is a habitat for commensal organisms and, from the perspective of the immune system, in constant contact with foreign antigens and the environment [107]. Tregs, making up approximately 10% of resident T cells in the skin, is essential in regulating the complex immune interaction and homeostasis needed for defense, tolerance, and tissue repair [108]. Dysregulation of Tregs has been shown to contribute to many skin pathologies, such as psoriasis, contact dermatitis, pemphigus vulgaris, alopecia, and systemic sclerosis [109,110]. Animal models have shown that colonization of skin bacteria during neonatal development, but not in adulthood, can activate antigen-specific Tregs across an intact skin barrier, suggesting a window period crucial for commensal tolerance [111]. Cutaneous Tregs were found to express high levels of CD25, L-selectin, GITR, FOXP3, and intracellular CTLA-4, low levels of CD69, and high levels of the skin-homing molecules CLA, CCR4, and CCR6 [112]. The lack of homing molecules CD103, CCR4, or P- and E-selectin ligands impaired the migration and retention of Tregs within the skin and resulted in skin-specific autoimmunity [108]. Skin Tregs were found to be capable of expansion in both an antigen-specific and antigen-independent manner in response to dermal fibroblasts [112]. Depletion of Tregs was shown to result in the activation of dermal fibroblasts and activation of profibrotic gene expression in the skin, suggesting its role in fibrotic diseases [113]. The survival of skin memory Tregs was found to be dependent more heavily on IL-7 than on IL-2 [114]. The suppressive mechanism is thought to be mediated by the cytokines IL-10 and TGF-β under some circumstances but not others [108,112]. A unique function of skin Tregs is their role in hair growth. Hair follicle (HF) stem cells failed to transition into the active growth phase upon Treg depletion in animal studies. Skin Tregs apparently localize next to HFs, and expression of the Notch ligand Jagged-1 (Jag1) on Tregs facilitate the proliferation of HFs [115]. This not only facilitates hair growth but also facilitates the migration of HF stem cells into a wound, where they differentiate toward keratinocytes in the context of skin injury [27].

Although the skin is susceptible to several primary sun-associated cutaneous malignancies, it is generally not a site of metastatic disease for other cancers. Metastatic cancer has been found in only 0.7–9% of cases, with lung and breast carcinomas being most the common cancers of origin [116]. Consistent with the theory that Tregs participate in a natural feedback response to T cells sensitive to cancer neoantigens, melanoma metastasis that contains infiltrating activated CD8 T cells has the highest expression of IDO and Tregs [117]. Similarly, activated OX40^+^ Tregs are found within cutaneous squamous cell carcinomas and are associated with subsequent metastases [118]. More Tregs have been found in specimens taken from the head and neck than in those taken from both the trunk and extremities. The head and neck are affected most frequently by cutaneous metastasis per unit surface area. Given that skin Tregs preferentially concentrate near HFs, the authors speculated that areas of increased HF density, such as the scalp, may be more permissive to tumor growth [119]. Mechanistically, the expression of the mitochondrial enzyme arginase 2 (ARG2) was preferentially increased in Tregs in metastatic melanoma compared to other diseases, such as psoriasis. Experimentally, the overexpression of ARG2 suppressed mTOR signaling, enhancing Treg fitness, while inhibition of ARG2 decreased Treg accumulation in tissues and suppressed Treg activation [120]. In a mouse model, the increased invasive and metastatic potential of melanoma was found to be mediated by direct contact between melanoma cells and Tregs. Elevated expression of TGF-β by Tregs induced the epithelial-to-mesenchymal transition (EMT), contributing to increased lung metastasis [121]. Interestingly, the tumor-infiltrating Treg quantity in mucosal melanoma was significantly higher than that in cutaneous melanoma and was associated with a reduced response to immunotherapy, raising the possibility that despite the common presence of Tregs, the skin may not be a tissue that favors the suppression of antitumor immunity [122]^.^

## Tregs in LNs metastasis

LNs are ubiquitous throughout the body and adjacent to every organ. They are part of the lymphatic system and are essential for immunosurveillance and the control of autoimmunity. They are sites of lymph drainage from all tissues, where organ-specific environments and their associated antigens are presented for lymphocyte priming. Therefore, LNs can be considered sentinel and reservoir sites that provide additional adaptive immune cell support to tissues in need of pathogenic defense [123]. However, autoimmunity can arise when dysregulated recognition of self-antigens by adaptive immune cells leads to priming and activation of T follicular helper cells (*T*_FH_) and autoreactive B cells in LN germinal centers (GSs) [124]. Tregs are critical in LNs at steady-state and maintain a delicate balance between immunity and tolerance. Experiments with murine models have shown that LN stromal cells induce Treg activation in an IL-2-dependent manner [124]. LN-specialized T follicular regulatory (Tfr) cells may play a dominant role in controlling T_FH_ and B cells in LNs and are characterized by higher expression of ICOS, PD-1, CXCR5, TGF-β, and PD-1 [125].

LNs are frequent sites of metastatic spread; therefore, interest in studying the roles of Tregs in LN metastases remains high. Studies of the physiological trafficking of Tregs have demonstrated that Tregs with an effector phenotype, such as CD103-expressing Tregs, are preferentially retained in LNs, compared to other CD4 T cells [126,127]. There is evidence that an increased number of Tregs is associated with LN metastases in multiple tumor types. In a study of 30 patients with lung adenocarcinoma, Treg levels were elevated in LNs with metastatic tumor foci involvement but not in benign LNs, suggesting a critical role for Tregs in the formation of an immunosuppressive TME [128]. Studies of breast cancer surgical specimens suggested that tumor invasion into draining LNs is associated with Treg accumulation [129]. In a recent study by Gonzalo Nunez et al., Tregs from matched tumor-invaded and noninvaded tumor-draining LNs were compared with those in primary breast tumors, and the results demonstrated that Treg frequency increased with nodal invasion. Tregs also express higher levels of coinhibitory/stimulatory receptors than effector cells [129]. In patients with colorectal cancer, a higher proportion of Tregs and other T cells with suppressive immunophenotypes were found in regional LNs, especially those nearest a tumor [130].

## Tregs in the brain and adrenal metastasis

The brain was originally considered an “immune-privileged” organ site, initially defined through early experiments showing that the blood-brain barrier limited access to immune cells circulating in the peripheral immune system, an effective lymphatic system to drain the CNS was lacking, and APCs that express MHC class I and II molecules were absent [131]. However, over the past two decades, substantial progress has been made in understanding neuroimmune interactions, and the brain is currently regarded as an organ that has evolved with complex and dynamic immunoregulatory functions [132]. Due to its unique anatomic location and spatial constraints, the brain requires potent immune regulation, as an overreactive immune response can cause immediate and life-threatening CNS damage [133,134]. Tregs in the brain appears critical for mediating immune homeostasis and facilitating injury repair. In the brain, Tregs appear to be activated by microglial cells, which trigger the secretion of the suppressive cytokines IL-10 and IL-35 [132]. Multiple sclerosis (MS) is an autoimmune inflammatory disorder of the CNS, and Treg dysregulation and related genetic polymorphisms have been associated with MS. For example, reduced IL-10 production, genetic variations of CD25, reduced CTLA-4 and TGF-β expression, and fewer suppressive Tregs in the CNS have been reported in patients with MS [135,136]. In experimental autoimmune encephalomyelitis (EAE) animal models, antigen-specific Tregs mediated the control of autoimmune pathology [137]. Regarding tissue repair, ablation of Tregs promoted the worsening of infarcts in a brain ischemia model, and transfer of IL-10-deficient Tregs was ineffective in secondary prevention of infarct growth, suggesting the importance of Treg production of IL-10 [138]. IL-33 production by stromal cells and ST2 expression on Tregs were shown to be required for Treg trafficking to injured sites in the CNS [139]. Interestingly, brain Tregs express the serotonin receptor 5-HT_7_ and respond to neurotransmitters by upregulating suppressive and pro-repair molecules such as AREG [139].

Treatment of brain tumors and metastases without overstimulating local immunity within the brain is paramount to its safety and success [133,134]. In metastatic melanoma with brain metastasis, CPI is effective but has significantly lower response rates than cutaneous lesions, suggesting an influence of tissue-specific immunoregulatory factors [140]. Although the mechanisms of resistance may be different between metastatic and primary brain tumors, for the latter, CPIs have not achieved similar efficacy [141]. Tregs were found to infiltrate brain tumors in high numbers and to be increased in the blood and tumors of glioma patients and murine models, and the depletion of Tregs has been associated with improved survival [142,143]. In lung cancer patients with brain metastasis, increased Tregs were found in the peripheral blood and were associated with increases in MDSCs and IL-6 expression [143]. Wainwright et al. reported Helios^+^ thymus-derived natural Tregs to be the predominant type of Tregs infiltrating brain tumors [144]. The meningeal lymphatic vessels provide drainage from the CNS into the cervical LN, where brain tumor-associated Tregs are found [145]. Several groups have reported that the TGF-β and IDO pathways are mechanistically relevant [146,147]. Gliomas express high levels of TGF-β, and TGF-β neutralization leads to decreases in brain tumor-infiltrating Tregs, suggesting a role for TGF-β in modulating Tregs [148]. IDO is also elevated in glioma specimens, and its upregulation has been associated with a decrease in OS. In a model of brain tumors, IDO-competent tumors were accumulated and support Treg expansion in IDO-deficient mice, suggesting the importance of IDO as a local factor modulating Treg activity [147].

The adrenal gland is the primary site of endogenous glucocorticoid production, and given that glucocorticoids are associated with tolerogenic T cell activity and reduced immunotherapy response, there is a possibility that the adrenal microenvironment is uniquely immunosuppressive [149]. Tissue-specific Treg biology in the adrenal gland is not well understood; however, in the context of glucocorticoids, it has been shown that Tregs express β1-adrenergic and glucocorticoid α receptors; therefore, these cells may be susceptible to steroid modulation [150,151]. In stress models, glucocorticoid signaling has been reported to decrease systemic Treg levels or disrupt their function [150,151]. Given the unique function of the adrenal gland as the source of glucocorticoids and the known deleterious effects on both effector T cells and Tregs, more research is needed to understand the potential influence of Tregs in the adrenal gland in the context of cancer immunotherapy. Indeed, adrenal tumors are heavily infiltrated by Tregs, and a recent analysis of CPI trials with adrenal cancers revealed a generally dismal response [152]. Even for MSI-H mCRC patients where CPI is extremely effective, emerging reports indicate that the adrenal site may be a sanctuary site of resistance showing a dissociated response pattern [153].

## Systemic and tissue-specific Treg targeting for cancer immunotherapy

Given the importance of Tregs in promoting tumor growth and metastasis, there has been great interest in developing immunotherapies that target Tregs in cancer. The role of Tregs in antitumor immunity was initially reported in 1999 by Sakaguchi et al. [154]. In this study, the group demonstrated increased tumor rejection in mice treated with anti-CD25 antibodies, which depleted CD4^+^CD25^+^ Tregs in mice. Similarly, in other preclinical models, depletion of Foxp3^+^ Tregs was effective not only in treating tumors but also in preventing malignant formation, acting as a cancer vaccine [155].

Despite the promising preclinical results obtained over decades, the clinical application of targeted Treg therapy has been difficult as cancer immunotherapy. First, Tregs and activated effector T cells share many of the same cell surface markers, such as CD25 and CTLA-4, making it challenging to specifically deplete Tregs without affecting effector T cells. Second, Tregs also play an indispensable role in preventing autoimmunity, and thus far, it has been challenging to specifically deplete tumor-directed Tregs without affecting peripheral Tregs (e.g., circulating Tregs, lymphoid-resident Tregs, and tissue-resident Tregs) to prevent autoimmune side effects. To overcome these issues and achieve greater specific antibody-mediated killing of tumor Tregs, one approach is to better target surface molecules that are expressed or are elevated only on intratumor Tregs, such as CD25, CTLA-4, GIRT, 4-1BB, OX-40, LAG3, TIGHT, CCR4, and CCR8 [156]. Antibody-dependent Treg cell depletion can exploit differences between tumor Treg and effector T cell kinetics and targets. While there are many similarities in the cell surface markers expressed on Tregs vs. effector T cells, there are differences in the expression levels and kinetics currently being studied, which can be exploited. For example, an anti-CTLA-4 monoclonal antibody within the IgG2a subclass depleted Tregs specifically in tumor tissue and thus enhanced antitumor immunity [157]. Tregs constitutively express CTLA-4, but conventional T cells express only CTLA-4 when they are activated, and conventional T cells express it at much lower levels than Tregs; thus, it is possible to engineer antibodies with Fc regions specifically designed to deplete Tregs without significantly reducing the effector T cell population [158]. An anti-CCR4 monoclonal antibody with high antibody-dependent cell-mediated cytotoxicity (ADCC) was used to deplete Tregs and enhance antitumor responses [159]. Similarly, monoclonal antibodies against CD25, OX-40, and GITR enhanced ADCC-mediated Treg depletion and slowed tumor growth [160–162]. Thus, when tissue-specific modulation is taken into account in greater detail, surface proteins expressed at high levels on intratumor Tregs can be targets of greater tumor Treg depletion by ADCC monoclonal antibodies.

In addition to exploiting differences in intratumor Treg surface protein kinetics, the specificity of Treg deletion can be confined to tumor tissue using other novel approaches. Several techniques have been employed to generate local Treg depletion. First, local intratumoral injection of anti-CTLA-4 antibody [163,164] or anti-GIRT mAb [165], not systemic delivery, has led to decreases in tumor size while inducing tumor regression at distant sites with few systemic effects. Another technique involves the use of an antibody conjugated with a photoactivatable dye to induce lethal damage to the cell membrane of the target cells upon near‐infrared (NIR) light exposure. Using a mouse model, Sato et al. demonstrated that an anti-CD25 mAb conjugated to a photoactivatable dye deleted only tumor-localized Tregs upon exposing just the tumor to NIR light [166]. Chimeric antigen receptor (CAR) T cells engineered to deliver cytokines such as IL-12 specifically to the TME, termed “armored CARs,” showed antitumor efficacy that included resistance to Treg suppression at the tumor site [167]. Finally, a promising strategy involves blocking the migration of Tregs to the TME. In a melanoma study, CCR4 was required for the homing Tregs to nascent tumor sites from LNs, and BRAF^V600E^ signaling controlled the expression of CCR4 chemokines, raising the possibility that inhibiting this signaling with BRAF inhibitors may have reduced Treg recruitment to the tumor [168]. In patients with cutaneous T cell lymphoma, mogamulizumab, a defucosylated anti-CCR4 antibody, reduced the levels of CCR4^+^ Tregs and showed significant efficacy [169]. Thus, this site-specific antibody activation, migration, and cellular delivery strategies enable the depletion of intratumoral Tregs or suppression of their immune-suppressing activity with a diminished effect on peripheral Tregs, potentially allowing improved therapeutic efficacy with distinct cancer immune responses without the induction of autoimmunity.

Another emerging area of interest is combining locoregional metastatic-site interventions with CPIs to induce an augmented response. Although the abscopal effect, or treatment response at a tumor site that was not the direct target of local therapy, has been sporadically reported, these effects have not been widely studied in the era of modern immunotherapy or with improved technology, which enables an analysis of tissue-specific regulatory mechanisms in detail. Locoregional cancer treatment therapies, including radiotherapy and surgery, combined with CPIs, are readily available and rational approaches for removing tumors and reducing tissue-resident suppressors such as Tregs to tip the immune balance toward antitumor immunity, potentiating the abscopal effect. Although clinical data on surgical metastasectomies in combination with CPIs are not yet available, experimental models have shown that surgical removal of metastatic tumors can eliminate immunosuppressive elements and is a powerful local force that disrupts the tumor-immune ecosystem [170]. However, the subsequent redistribution of immune cells following surgery may swing immunosuppressive responses in cancer patients in either direction. Surgical trauma and wound healing responses following surgery can counterbalance the potential benefits of surgery. Krall et al. [171] developed a mouse model that showed that activation of wound repair mechanisms induced by surgical incisions resulted in diminished local and systemic antitumor immunity. Finally, laparotomy in preclinical models has been shown to induce CCL18 expression within the peritoneal cavity and promote Treg recruitment [172]. Thus, while randomized trials are ongoing to examine whether surgeries such as palliative nephrectomies may benefit therapeutic antitumor immunity (NCT03977571), there is a conceptual concern that surgery-related immunosuppression may limit this approach [173].

Radiotherapy is a noninvasive locoregional treatment modality. While palliative fractionated radiation has historically been used only for symptom palliation in metastatic cancer patients, there is emerging random evidence indicating that consolidation of disease in oligometastatic patients with focal stereotactic ablative body radiation may improve the progression-free and OS of patients [174]. Data obtained to determine whether radiotherapy alters Tregs are controversial. Chemoradiation has been shown to increase Tregs in head and neck cancer patients [175] but has also been shown to decrease Tregs in rectal cancer and cervical cancer patients [176] and pancreatic cancer patients [177]. Thus, it is not yet fully understood whether ablative consolidation with radiotherapy alters Tregs to enhance therapeutic antitumor immunity in patients, and additional understanding of tissue-specific immunology and the clinical correlation is required.

In preclinical models, it has been shown that fractionated large-field or whole-body radiotherapy actually increases the number of systemic Tregs [178]. In multiple preclinical models, radiation can cause lymphopenia, which can promote relative expansion in Treg cell numbers because they are more radioresistant than other lymphocytes [179]. Preclinical evaluation of the impact of radiation on Tregs has indicated that the dose and degree of fractionation matter and can lead to diverse immune responses. Ablative hypofractionated and fractionated radiation reduces Treg numbers in MC38 models [180]. This finding has also been observed in LM8 osteosarcoma models [181]. Mechanistically, radiotherapy has been shown to diminish tumoral CCL22, resulting in a diminished number of Tregs [182]. In contrast, subablative doses of radiation increased the number of intratumoral Tregs in multiple models [183]. Although radiation can induce TGF-β expression, which pleiotropically promotes Tregs [184,185], radiotherapy can also induce Treg expression in TGF-β-independent pathways [186]. In terms of the abscopal effect, there are emerging preclinical data suggesting that radiotherapy may promote systemic antitumor immune responses and promote tumor rejection of unirradiated tumors [187,188]. However, currently, evidence showing that radiotherapy promotes systemic antitumoral immune responses in unirradiated tumors in patients is limited [189]. Hence, there is significant interest in developing combinatorial strategies to enhance systemic antitumor immune responses that involve Tregs. Depletion of Tregs using genetic approaches promotes antitumor immunity following irradiation of preclinical models of mesothelioma [190]. Similarly, GITR agonizts and PI3Kαδ inhibitors that deplete Treg numbers promoted the antitumor efficacy of radiation exposure in multiple models [191,192]. Finally, inhibition of TGF-β in concert with radiotherapy at low doses can modulate antitumor immune responses and may diminish the effect of Tregs in patients [185]. Collectively, these data suggest that the degree to which radiation exposure modulates Tregs depends on the context and intensity of the therapy, which may depend on tissue-specific factors. Future studies will provide clinical and mechanistic insights into how to precisely combine radiotherapy with immunotherapy to enhance overall antitumor immunity.

## Conclusion

Cancer immunotherapy drugs such as CPIs provided the first proof-of-principle suggesting that we can modulate antitumor immunity to cure metastatic cancer. However, most patients do not respond to CPIs, and there is substantial variability in efficacy across tissue sites, which is observed not only in primary tumors but also at metastatic sites. To extend the benefit of these drugs to include more than a minority of cancer patients, a more complete understanding of tissue-specific Tregs is needed. Over the past several decades, in addition to their roles in cancer, Tregs have been studied as critical regulators of the immune response in tissue repair, peripheral tolerance, allergy, inflammation, and interactions with the commensal microbiome [193]. However, despite our current knowledge, the successful therapeutic manipulation of Tregs has been difficult. There is growing consensus that to overcome the remaining challenges, we must reconcile the variability of the responses distinct to cancer types and organ metastasis with the specialized, tissue-adapted mechanisms of Treg immunoregulation. The observation that not all tissue sites respond equally to immunomodulating drugs coupled with recent advances revealing numerous tissue-specific adaptations of Tregs, suggests that tissue-specific targeting of Tregs may be necessary to overcome their regulatory control. Environmental inputs affecting Tregs, such as oxygen tension changes in the lungs, tissue regeneration in the liver, changes in the gut microbiome, bone turnover, and even neuronal signals in the CNS, can have profound context-specific effects on tissue-resident Tregs and impact tumor immunity. In addition, since Tregs are essential for maintaining immune homeostasis, safe and effective immunotherapy will require more precise targeting of Tregs, not complete systemic deletion. Insights into specialized Tregs suggest that this specific targeting be achieved by exploiting differences in cell surface receptors, genetic programming, the kinetics of protein expression, and other context-specific local mediators (Fig. 2). To enhance both the efficacy and safety of combined precision immunotherapy, treatments can be tailored to specific primary or metastatic sites with locoregional interventional approaches or with drugs designed to be deleterious to tissue-specific Tregs by targeting pathways most critical to the relevant Treg biology. Decades of research on Tregs point to their critical importance on the roadmap to a cure for metastatic cancer and have paved the way for the future of precision cancer immunotherapy.
